# *Phytophthora parasitica*: a model oomycete plant pathogen

**DOI:** 10.1080/21501203.2014.917734

**Published:** 2014-05-19

**Authors:** Yuling Meng, Qiang Zhang, Wei Ding, Weixing Shan

**Affiliations:** a State Key Laboratory of Crop Stress Biology for Arid Areas, College of Plant Protection, Northwest A&F University, 3 Taicheng Road, Yangling, Shaanxi 712100, China; b College of Plant Protection, Southwest University, Beibei, Chongqing 400715, China

**Keywords:** oomycete, *Phytophthora parasitica*, model pathosystem, tobacco, *Arabidopsis thaliana*

## Abstract

Oomycetes are eukaryotic microorganisms morphologically similar to but phylogenetically distant from true fungi. Most species in the genus *Phytophthora* of oomycetes are devastating plant pathogens, causing damages to both agricultural production and natural ecosystems. Tremendous progress has been achieved in recent years in diversity, evolution and lifestyles of oomycete plant pathogens, as well as on the understanding of genetic and molecular basis of oomycete-plant interactions. *Phytophthora parasitica* is a soilborne pathogen with a wide range of host plants and represents most species in the genus *Phytophthora*. In this review, we present some recent progress of *P. parasitica* research by highlighting important features that make it emerge as a model species of oomycete pathogens. The emerged model pathogen will facilitate improved understanding of oomycete biology and pathology that are crucial to the development of novel disease-control strategies and improved disease-control measures.

## Introduction

Oomycetes represent a group of eukaryotic microorganisms related to diatoms and brown algae and cause many destructive diseases to plants and animals. Among this group, the genus *Phytophthora* includes over 100 species (Kroon et al. 2012), and the number is increasing. *Phytophthora* species, the plant destroyers, have been a great threat to agricultural production and natural ecosystems. A notable example is *Phytophthora infestans*, the pathogen of the potato and tomato late blight, which triggered the Irish Famine in the 1840s but remains to be a difficult disease to control worldwide (Haas et al. 2009). Also, the recently emerged *Phytophthora ramorum* caused the Sudden Oak Death in 1990s and severely damaged woodlands in North America and Europe (Grünwald et al. 2012).

Oomycetes have several important characteristics distinct from true fungi (Latijnhouwers et al. 2003). For example, oomycetes are diploid while fungi are haploid, oomycete hyphae are nonseptate and multinucleated while fungi hyphae are septate. Many oomycetes are sterol aux-otrophs. Great differences in cell wall composition between oomycetes (consist mainly of *1,3-b-glucans*, some 1,6-*b*-glucans and 1,4-*b*-glucans) and fungi (mainly of chitin) are notable (Werner et al. 2002; Latijnhouwers et al. 2003). The majority of fungicides target chitin and sterol synthesis and are ineffective for the control of oomycete diseases.

Understanding the mechanisms of oomycete pathogenicity is essential to develop disease-control measures. Most researches have focused on a few species, particularly *P. infestans, Phytophthora sojae* and *Hyaloperonospora arabidopsidis. P. infestans* is a foliar pathogen capable of infecting potato and tomato, *P. sojae* infects soybean *{Glycine max*) only. *H. arabidopsidis* is an obligate biotrophic pathogen of the model plant *Arabidopsis thaliana*.

**Figure 1.**
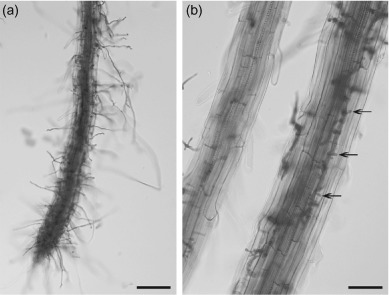
Root infection of *Arabidopsis thaliana* by *Phytophthora parasitica*, (a) Heavy colonization of root tissues by *P. parasitica*. Scale bar = 100 µm. (b) Numerous haustoria-like structures (Ha) developed (arrow). Scale bar = 50 µm.

Compared with these, *Phytophthora parasitica* Dastur (syn. *Phytophthora nicotianae* Breda de Haan) is a typical root pathogen with broad host ranges, being capable of infecting over 72 plant genera (Hickman 1958). With over 100 species in the genus *Phytophthora*, the features of the pathogen represent majority of *Phytophthora* species (Kroon et al. 2012). In recent years, many research have focused on this species and extensive molecular genetic tools and genomics resources have been developed. Especially, the pathosystem of compatible interaction (Figure 1) between *A. thaliana* and *P. parasitica* has been established. The development of the model pathogen is expected to facilitate accelerated understanding of oomycete pathogenesis, by accessible numerous genetic and genomics resources and associated tools with the model plant *A. thaliana*, which will ultimately lead to the development of novel disease-control strategies and improved disease-control measures.

## Phylogeny of *Phytophthora parasitica*

Oomycetes belong to the kingdom Stramenopila, which also includes brown algae and diatoms. Of all oomycetes, *Phytophthora* is the best-studied genus. Using a genus-wide phylogeny analysis, 116 *Phytophthora* species are divided into 10 clades within the genus (Kroon et al. 2012). *P. parasitica* is classified in the *Phytophthora* clade 1 and its closest relatives include *P. infestans*. There are three divisions in this clade (a, b, and c). *P. parasitica* is singular in this clade, because it could not be placed in one of the subclades based on sequence analysis (Blair et al. 2008; Kroon et al. 2012). The phylogenetic relationships in the genus may serve as an inspiration to help understand more about *Phytophthora* species.

## The biology and life cycle

**Figure 2.**
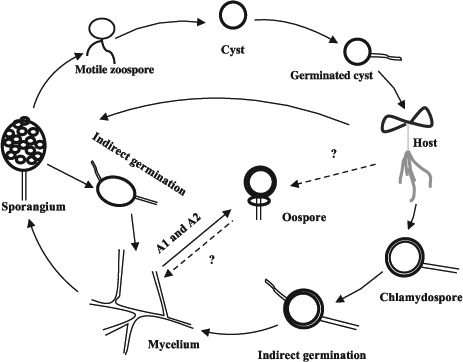
The life cycle of *Phytophthora parasitica*.

The typical *Phytophthora* life cycle includes both asexual and sexual phases. The life cycle of *P. parasitica* is shown in Figure 2. The hyphae are hyaline and aseptate, sometimes with hyphal swellings. *P. parastica* produces asexual sporangia, zoospores and chlamydospores. The sporangia are difficult to be released from the hyphae, which is different from the released, airborne sporangia of *P. infestans*. Zoospores are produced by the sporangia and are wall-less cells with two flagella, which enable them to swim. Chlamydospores are thick-walled, multinucleated asexual spores, usually produced at the tips or in the middle of hyphae. *P. parasitica* is predominantly het-erothallic, requiring A1 and A2 mating types for the production of oospores (Ko 1981).

Efficient spore production and dispersal are essential for successful infection (Latijnhouwers et al. 2003). Zoospores are considered to be the major infective agents that initiate plant diseases for most *Phytophthora* species. Once reached the plant surface, zoospores become immobile cysts, subsequently germinate. *P. parasitica* infects roots and leaves by producing a specialized structure, appressorium, formed at the tip of germ tubes (Kebdani et al. 2010; Wang et al. 2011). Then, invasive hyphae are developed and haustoria-like structures are formed. At last, abundant sporangia formed on the surface of infected plants. If the environmental condition is unfavorable, chlamydospores are produced from the hyphae. The chlamydospores can survive in soil for several years, and serve as the primary inoculum in the field (Van Jaarsveld 2001). Sexual reproduction is an efficient and important way for the pathogen to produce genetic variation, including potentially large number of various genotypes and pathotypes that enable the pathogen to adapt to unfavorable conditions, particularly the introduction of new resistant genotypes of the host plant. However, to which extent the sexual reproduction play a role in *P. parasitica* remains unclear.

## Genetic manipulation

Genetic manipulation is essential for analysis of gene functions in a given organism. To date, genetic transformation remains difficult for oomycetes, only reported to be successful for few species at variable but low efficiencies compared to true fungi. There are four methods developed for transforming foreign DNA into the genome of *Phytophthora*. The polyethylene glycol (PEG)-mediated transformation protocol was developed by Judelson et al. (1991), the first description of reliable method for transformation in an oomycete pathogen. Subsequently, the PEG transformation method was successfully used in several other species including *P. sojae* (Judelson et al. 1993), *Saprolegnia monoica* (Mort-Bontemps and Fvre 1997), *P. parasitica* (Bottin et al. 1999), *Phytophthora palmivora* (van West et al. 1999b), *Phytophthora brassicae* (Si-Ammour et al. 2003), *Pythium aphanidermatum* (Mcleod et al. 2008). Alternative transformation procedures were developed, such as particle bombardment in *P. infestans* (Cvitanich and Judelson 2003), *Agrobacterium tumefaciens*-mediated transformation *in P. infestans* (Vijn and Govers 2003) and electroporation of zoospores in *Phytophthora capsici* (Huitema et al. 2011), for manipulating gene expression levels.

**Figure 3.**
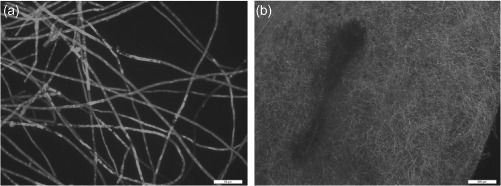
Cytological characterization of *Phytophthora parasitica* transformant and the infected leaf of *Arabidopsis thaliana*. (a) Hyphae of *P. parasitica* transformant expressing GFP. Scale bar = 50 μm. (b) Heavy colonization on leaf tissue of ecotype Col-0. Scale bar = 500 μm.

This PEG-mediated transformation method has been successful in *P. parasitica* (Bottin et al. 1999). The *P. parasitica* transformants expressing green fluorescent protein (GFP) were used for cytological analysis of the colonization in the host plant tomato (Le Berre et al. 2008; Kebdani et al. 2010) during infection, and for analysis of the potential and application of host-induced gene silencing in *P. parasitica* (Zhang et al. 2011). The *P. parasitica* expressing GFP is very stable and can be successfully used for assessment of susceptibility of the host plant to the pathogen (Figure 3).

With the development of genetic transformation method, pathogens were labeled with fluorescent proteins and have successfully been employed to help answer questions about how the pathogens interact with the host plants. For an excellent example, Whisson et al. (2007) used *P. infestans* transformants expressing GFP and translational fusions of effector Avr3a with the monomeric red fluorescent protein to define that Avr3a is secreted from haustoria and translocated into the host. Ah-Fong and Judelson (2011) labeled various organelles in *P. infestans* and provided a series of vectors designed for expressing different fluorescent proteins.

Due to diploid nature and lack of homologous recombination-based gene disruption in oomycetes, RNA silencing emerged to be an important approach for downregulation of target genes in *Phytophthora*. Internuclear gene silencing was reported in *P. infestans* (Kamoun et al. 1998; van West et al. 1999a). The introductions of sense, antisense, and hairpin constructs were all subsequently confirmed to induce gene silencing, enabling gene function studies in *Phytophthora* (Ah-Fong et al. 2008). Using *inf1* as a target, Ah-Fong et al. (2008) compared three method including PEG treatment of protoplasts, zoospore electroporation, and microprojectile bombardment and they found that hairpin vectors combined with protoplast transformation was the highest to silence genes. Gene silencing has been used to analyze a number of genes in oomycete, including *P. parasitica*. For example, the suppressed expression of cellulose-binding elicitor lectin (CBEL) in transgenic strains of *P. parasitica* caused severe impartation in adhesion of the pathogen to the cellophane membrane, differentiation of lobed structures in contact with cellophane, and formation of branched aggregating hyphae (Gaulin et al. 2002). The transformants silenced with *PnDLCl* in *P. parasitica* (Narayan et al. 2010) released nonflagellate, nonmotile zoospores from their sporangia. High level (more than 80% reduction) of *PnPMA1* silencing in *P. parasitica* led to the production of nonflagellate and large aberrant zoospores, rapid transition from zoospores to cysts, and a decreased germination rate of cysts, indicating that *PnPMAl* plays important roles in zoospore development (Zhang et al. 2012).

## Genomics

A large-insert bacterial artificial chromosome (BAC) library (Shan and Hardham 2004) using nuclear DNA from *P. parasitica* was constructed. The library contains 10,752 clones with an average insert size of 90 kb and is free of mitochondrial DNA. The genome size of *P. parasitica* was estimated to be 95.5 Mb by the analysis using several DNA probes and physical mapping. The BAC library provides important resource for genome analysis of genes of *P. parasitica* and is useful for the genome reconstruction.

Expressed sequence tags (EST) analysis is a useful approach to get understanding of the basic biology and interaction with host plant of pathogen. Several EST libraries have been generated from different stages including mycelium (Panabières et al. 2005), zoospores (Škalamera et al. 2004), germinated cysts (Shan et al. 2004b), penetration process (Kebdani et al. 2010), late infection stage (Le Berre et al. 2008), and other culture conditions (Rosa et al. 2007) of *P. parasitica*. The EST resources of different developmental stages of *P. parasitica* were shown to be important for the identification of specific pathogenicity-related genes, for example, over 300 clones representing 146 unigenes were identified by upregulated expression in germinated cysts (Shan et al. 2004b), more than 400 clones representing 240 genes were shown to be preferentially expressed in zoospores (Škalamera et al. 2004) in the two small EST collections, and 60% of the appressorium-derived sequences were not present in other *P. parasitica* EST collections (Kebdani et al. 2010). Sequencing of two infection libraries of different stages showed about 9% (147/1689) and 2% (42/2022) of *P. parasitica* unigenes, respectively, that gave no significant hits in the genome sequences of *P. infestans, P. sojae*, and *P. ramorum* (Le Berre et al. 2008; Kebdani et al. 2010). Data from the ESTs of different infection and development stages assist to provide information for future functional analysis and to understand the genetics and physiology of *P. parasitica*.

The draft genome sequence of *P. parasitica* has been completed (https://olive.broadinstitute.org/projects/phytophthora_parasitica). The genome sequence of *P. parasitica* includes about 23,121 predicted genes within the 82-Mb genome compared to 18,178 genes for the 240-Mb genome of *P. infestans*, 16,988 genes for the 95-Mb genome of *P. sojae* (Judelson 2012). The number of predicted genes is more than the two narrow host range species *P. infestans* and *P. sojae*, implicating possible relation to its capability to infect large number of plant species. The *P. parasitica* genome project also involves sequencing of multiple isolates isolated from diverse host plants and geographic distant locations, presumably with diverse genetic backgrounds. One of the goals is to make a comparative genomic study to identify genes that determine host range in *P. parasitica*.

By now, genome sequences have been available for several oomycetes including *P. capsici* (Lamour et al. 2012), *Phytophthora cinnamomi, P. infestans* (Haas et al. 2009), *P. parasitica, P. ramorum* (Tyler et al. 2006), *P. sojae* (Tyler et al. 2006), *Pythium ultimum* (Lévesque et al. 2010), *H. arabidopsidis* (Baxter et al. 2010), *Albugo laibachii* (Kemen et al. 2011), and *Saprolegnia parasitica* (Judelson 2012). The sequenced oomycete pathogens genome size is from 37-Mb to 280-Mb and the predicted gene contents range from about 13,000 to 26,000 genes (Judelson 2012). The huge resources of the genome sequences provide more information for answering the questions about the biology, evolution, and pathogenesis of oomycete pathogens. One example is that the sheer number of protein effectors has been uncovered, some of which are crucial for pathogenesis.

## The host specificity

*P. parasitica* is a species complex, capable of infecting numerous plant species, including model plants *Nicotiana tabacum* and *A. thaliana*. This allows development of tractable pathosystems for the study of the interaction between *P. parasitica* and host plants in the laboratory.

*P. parasitica* caused black shank disease of tobacco worldwide. It can attack all parts of tobacco including roots, stems, and leaves at any growing stages. As its name, the most common symptom of the disease is the black base or shank of the stalk. Infection is usually through the roots. The roots are initially water soaked then rapidly become necrotic. In young plants, the stems become brown to black and the darkening can extend up the stalk several centimeters down into root system, then the plants damp off. The pathogen can directly infect the leaves, brown-to-black or large circular lesions occur following wet weather. The disease can be devastating to tobacco in the greenhouse as well as in the field (Stavely 1979). The cytological observations between *P. parasitica* and tobacco have also been described (Benhamou and Côté 1992; Séjalon-Delmas et al. 1997; Bottin et al. 1999).

*P. parasitica* interacts with its host tobacco in a race-cultivar specific manner. Races are defined by the ability of the pathogen to infect various cultivars carrying different resistance genes. The knowledge of resistant resources of tobacco to *P. parasitica* is limited, mainly including the oligogenic black shank resistance of the cigar-wrapper *N. tabacum* cv. Florida 301, dominant and monogenic resistance from *Nicotiana plumbaginifolia* Viv and *Nicotiana longiflora* Cav, the cigar-type tobacco cv. Beinhart 1000-1, and the flue-cured cultivar Coker 371-Gold (Van Jaarsveld et al. 2002; Antonopoulos et al. 2010).

There are four races (race 0, 1,2, and 3) reported for *P. parasitica*. Race 0 is defined as being nonpathogenic on *N. plumbaginifolia* Viv while race 1 is pathogenic (Apple 1962). The evidence indicated that a single dominant gene controlled the resistance to race 0 (Goins and Apple 1970). Race 2 (Lamprecht 1973; Stavely 1979) was defined in South Africa by the differentiated reaction of tobacco ev. Delerest 202, which is resistant to race 2 but susceptible to race 0 and 1. Race 3 (Taylor 1975; McIntyre and Taylor 1978) can overcome resistance in cigar-wrapper tobacco and is tolerant to cold temperature.

The losses of black shank caused by *P. parasitica* are severe in worldwide tobacco production although many management programs including cultural practices, host resistance, and chemical treatments are deployed. Compared to other methods, the most effective approach to manage black shank is to use cultivars with high levels of resistance to all *P. parasitica* races. So there is a crying need for new resistance resources for *P. parasitica*.

## The model pathosystem

To better understand the interaction between a pathogen and its host, knowledge obtained from a good model pathosystem is necessary. Compared with the other host plant, *A. thaliana* is more efficient for accelerated understanding of *Phytophthora* biology and pathology. The fabulous wealth of *Arabidopsis*, including its genomic resources, mutant collections, natural ecotypes, and many associated genetic and molecular tools, allowed researchers to obtain fundamental knowledge on understanding molecular and cellular mechanisms in plants interacting with pathogens, notably the pathogen-associated molecular patterns (PAMPs) perception and PAMP-triggered immunity (PTI), nucleotide-binding and leucine-rich repeat domains (NB-LRR) based disease resistance and effector-triggered immunity (ETI), vesicle transport and polarized cellular defense responses, transcriptional output networks, and the interplay between disease resistance and hormone signaling pathways (Nishimura and Dangl 2010).

Attard et al. (2010) and Wang et al. (2011) described the compatible interaction between *A. thaliana* and *P. parasitica*, respectively. Cytological characterization showed that both the roots and leaves of *Arabidopsis* are susceptible to *P. paracitica* infection, as evidenced by development of water-soaked lesions, extensive pathogen colonization, and formation of abundant haustoria-like structures. The infection process is similar with the natural host, tomato (Le Berre et al. 2008). However, the disease severities were differential, dependent on the ecotypes of *A. thaliana* and strains of *P. parasitica*, indicating the presence of natural variation in host specificity between *A. thaliana* and *P. parasitica* (Wang et al. 2011). Moreover, the *A. thaliana* mutants with impaired salicylic acid (SA), jasmonic acid (JA), and ethylene (ET) signaling pathways are more susceptible than the wild type and also the transcripts for marker genes are transient accumulated (Attard et al. 2010). These results suggest that the JA, SA, and ET signaling pathways are all involved in the defense against *P. parasitica* which is different from three other described *Phytophthora* species that infect *Arabidopsis*, including *P. brassicae* (Roetschi et al. 2001; Schlaeppi et al. 2010), *P. cinnamomi* (Rookes et al. 2008), and *P. capsici* (Wang et al. 2013).

The model pathosystem was successfully used to answer interesting questions. For example, Zhang et al. (2011) examined the effect of transgenic expression of dsRNAs on the expression of homologous genes in the invading and colonizing oomycete pathogen. The results suggested that the oomycete pathogens might lack the genetic machinery required for uptake of external silencing signals, in particular dsRNAs, during biotrophic interaction. Diévart et al. (2011) used the pathosystem to analyze the expression of leucine-rich repeat receptor kinases (LRR-RK) genes, and the expression data suggest that oomycete LRR-RKs may play a role in several stages of the oomycete life cycle. Jaouannet et al. (2013) showed that a calreticulin from *Meloidogyne incognita* (Mi-CRT) plays an important role in infection success. They used the model pathosystem to test the susceptibility of stably transformed *A. thaliana* plants that express the secreted form of Mi-CRT to *P. parasitica*. Larroque et al. (2013) investigated the role of CBEL-triggered immunity, which benefit *A. thaliana* mutants and natural ecotypes, and also the specific interaction between *A. thaliana* and *P. parasitica*.

## The molecular basis of pathogenesis

The intimate attachment to host cells enables the parasitic pathogen to acquire nutrients easily; however, the relatively conserved components such as PAMPs or danger-associated molecular patterns from pathogens become stimuli of pattern recognition receptors-mediated resistance of plants. The successful pathogen is able to effectively evade or suppress PTI by secretion of a set of effectors, and initiates the disease in the plant. Some effectors could be specifically targeted by resistant proteins in plants and activates ETI (Jones and Dangl 2006).

In recent years, many researches have focused on effectors, which are key virulence factors of pathogens. Effectors are molecules and typically proteins secreted by the pathogen to manipulate host cell structure and function thereby facilitating infection and colonization (Kamoun 2006). Effectors can be classified in two groups according to their subcellular localization. The apoplastic effectors are released into the plant extracellular space and the cytoplasmic effectors are translocated inside the plant cell. Up to now, a number of effectors have been reported (Kamoun 2006), including enzyme inhibitors, small cysteine-rich proteins, Nepl-like family, two large classes of cytoplasmic effectors RXLR and CRN, as well as YxSL motif containing proteins in *P. ultimum* (Lévesque et al. 2010), and CHXC effectors in *Albugo* (Kemen et al. 2011). The described effectors of the pathogen play numerous and essential roles in the infection stage (Ali and Bakkeren 2011), such as disarming plant defense enzymes, suppressing host immunity or killing host cells.

The elicitors, like the elicitin gene *ParA1* (Kamoun et al. 1993), which are likely PAMP molecules, were identified and well characterized in *P. parasitica*. Other reported effectors include NEPl-Like protein NPP1 (Fellbrich et al. 2002), and the gene family encoding apoplastic polygalacturonases (Yan and Liou 2005; Wu et al. 2008). One of the apoplastic effectors is CBEL, firstly purified from *P. parasitica* cell wall. It induces strong defense reactions when infiltrated into leaf tissue of plant species including tobacco and *Arabidopsis* (Mateos et al. 1997; Séjalon-Delmas et al. 1997; Khatib et al. 2004) and it is necessary for the structure of the hyphal cell wall and attachment to cellulosic substrates such as plant surfaces (Gaulin et al. 2002). CBEL harbors a duplication of two types of domains (Tordai et al. 1999), and it has been detected in many oomycete species (Links et al. 2011).

Recently, a RXLR effector of *P. parasitica* PSE1, identified in a cDNA library for the penetrating stage of *P. parasitica* (Kebdani et al. 2010), was proved to favor the pathogen infection by modulating the auxin accumulation during the penetration process (Evangelisti et al. 2013).

Moreover, some RXLR effectors have been proven avirulence functions. Since the first oomycete *Avr* gene *Avr 1b* in *P. sojae* cloned (Shan et al. 2004a), a number of *Avr* genes have obtained, mainly from species of *P. sojae, P. infestans*, and *H. arabidopsidis* (Stassen and Van den Ackerveken 2011). However, little is known about the effector functions in *P. parasitica* and nothing about the *Avr* genes of this important pathogen, though host-genotype specificity in *P. parasitica* is notable.

Many methods have been designed to discover the effectors including Avr proteins, such as positional cloning, bioinformatic prediction, *in planta* expression, or some methods combined. Understanding what kind of effectors in *P. parasitica* and how effectors perturb host processes will be the major themes in the study of this pathogen.

## Summary

Driven by fundamental questions in oomycete evolution and pathology, *P. parasitica* emerged to be a model oomycete pathogen for understanding pathogenesis and host-pathogen interaction. The available genetic manipulation, abundant genetic and genomic resources of *P. parasitica* and its compatible interaction with the model plant *A. thaliana* will help understand fundamental questions, like the genetic basis of host range in the pathogen, effectors and their roles in pathogenesis, molecular dissection of effector function. The model study of *P. parasitica*, which represents the majority species of *Phytophthora*, will accelerate understanding of molecular plant-oomycete interactions and provide insight into novel disease-control strategies.
